# Regions of latest mechanical contraction correspond to regions of latest electrical activation: an electro-mechanical coupling study in patients undergoing cardiac resynchronization therapy

**DOI:** 10.1186/1532-429X-16-S1-P388

**Published:** 2014-01-16

**Authors:** Jonathan D Suever, Gregory Hartlage, Patrick Magrath, Michael Lloyd, John Oshinski

**Affiliations:** 1 Wallace H. Coulter Department of Biomedical Engineering, Georgia Institute of Technology/Emory University, Atlanta, Georgia, USA; 2 Department of Radiology and Imaging Science, Emory University School of Medicine, Atlanta, Georgia, USA; 3 Department of Medicine (Cardiology), Emory University School of Medicine, Atlanta, Georgia, USA

## Background

Numerous studies have shown that placing the left ventricular (LV) pacing lead in the latest contracting region (mechanically dyssynchronous) or the latest activated region (electrically dyssynchronous) of the LV improves patient response to Cardiac Resynchronization Therapy (CRT). However, no studies have examined electrical and mechanical dyssynchrony *in the same patients *to examine electrical-mechanical coupling. We hypothesized that the region of latest mechanical contraction would correspond to the region of latest electrical activation but the time between mechanical and electrical activation would vary between patients.

## Methods

Mechanical dyssynchrony: High temporal resolution steady-state free precession (SSFP) cine images were acquired with 60 frames per cardiac cycle in ten patients enrolled for CRT. The endocardial boundary was delineated in a stack of short-axis images. Radial contraction curves (RDCs) were generated for 100 locations on the endocardial boundary for each slice. The delay between each RDC and a patient-specific reference was determined using cross-correlation analysis. Mechanical delay times throughout the LV were mapped to the AHA 17-segment model. Electrical dyssynchrony: Regional electrical delay times were determined from local electrograms (EGMs) acquired during CRT device implantation. EGMs were acquired simultaneously from the RV lead (placed in the apex of the RV) and LV lead (placed at several sites in the free wall of the LV). 35 EGM recordings were obtained at several potential pacing sites in the 10 patients. The electrical delay was defined as the peak-to-peak difference between the EGMs from the RV and LV leads. Lead localization: Dual-plane fluoroscopic projection images were acquired at each LV lead recording site and was mapped onto the AHA 17-segment model. Mechanical delay times within a region the size of an AHA segment centered at the LV pacing location were averaged to obtain a corresponding mechanical delay time. Analysis: Mechanical delays (from MRI bullseye) and electrical delays (from EGM) were correlated on a patient-by-patient basis.

## Results

There was a strong linear relationship between electrical and mechanical delay times over all patients (R^2^=0.85 ± 0.21). The linear best fit in each patient yielded a positive slope (1.7 ± 1.3) and the slope varied greatly between patients (Range: 0.06-4.11). The site that exhibited the largest electrical delay always corresponded to the site of latest mechanical delay. The location of this latest activated/contracting site varied significantly between patients (Posterolateral: 3, Anterolateral: 3, Anterior: 1).

## Conclusions

The latest electrically activated site always corresponded to the latest mechanically contracting site; therefore either mechanical or electrically dyssynchronous regions can be targeted in an attempt to improve patient response to CRT.

## Funding

This study was funded in part by the National Science Foundation Graduate Research Fellowship Program.

**Figure 1:**
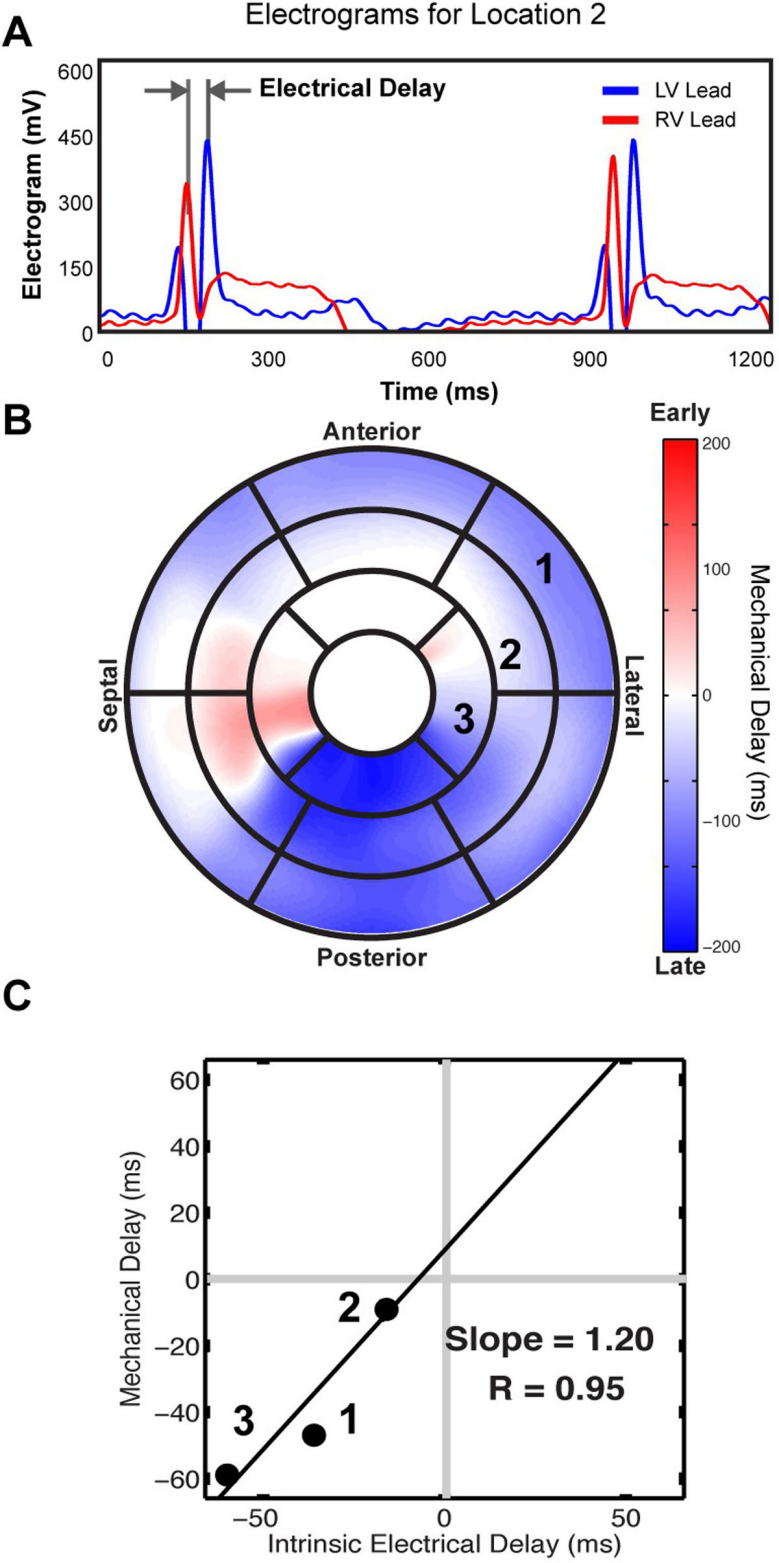
**Determination of Electrical and Mechanical Delay Times**: Electrical delay times were measured using peak-to-peak differences in the RV and LV EGMs (A). The measurement locations were mapped to the bullseye of mechanical delay times (B) to be compared (C).

